# Neurodynamic correlates for the cross-frequency coupled transcranial alternating current stimulation during working memory performance

**DOI:** 10.3389/fnins.2022.1013691

**Published:** 2022-10-03

**Authors:** Seong-Eun Kim, Hyun-Seok Kim, Youngchul Kwak, Min-Hee Ahn, Kyung Mook Choi, Byoung-Kyong Min

**Affiliations:** ^1^Department of Applied Artificial Intelligence, Seoul National University of Science and Technology, Seoul, South Korea; ^2^Biomedical Engineering Research Center, Asan Medical Center, Seoul, South Korea; ^3^Department of Electronics Engineering, Pohang University of Science and Technology, Pohang, South Korea; ^4^Laboratory of Brain and Cognitive Science for Convergence Medicine, College of Medicine, Hallym University, Anyang, South Korea; ^5^Institute for Brain and Cognitive Engineering, Korea University, Seoul, South Korea; ^6^Department of Brain and Cognitive Engineering, Korea University, Seoul, South Korea; ^7^Interdisciplinary Program in Brain and Cognitive Sciences, Korea University, Seoul, South Korea

**Keywords:** neuromodulation, nodal efficiency, transcranial alternating current stimulation (tACS), working memory, cross-frequency coupling (CFC)

## Abstract

Transcranial current stimulation is a neuromodulation technique used to modulate brain oscillations and, in turn, to enhance human cognitive function in a non-invasive manner. This study investigated whether cross-frequency coupled transcranial alternating current stimulation (CFC-tACS) improved working memory performance. Participants in both the tACS-treated and sham groups were instructed to perform a modified Sternberg task, where a combination of letters and digits was presented. Theta-phase/high-gamma-amplitude CFC-tACS was administered over electrode F3 and its four surrounding return electrodes (Fp1, Fz, F7, and C3) for 20 min. To identify neurophysiological correlates for the tACS-mediated enhancement of working memory performance, we analyzed EEG alpha and theta power, cross-frequency coupling, functional connectivity, and nodal efficiency during the retention period of the working memory task. We observed significantly reduced reaction times in the tACS-treated group, with suppressed treatment-mediated differences in frontal alpha power and unidirectional Fz-delta-phase to Oz-high-gamma-amplitude modulation during the second half of the retention period when network analyses revealed tACS-mediated fronto-occipital dissociative neurodynamics between alpha suppression and delta/theta enhancement. These findings indicate that tACS modulated top-down control and functional connectivity across the fronto-occipital regions, resulting in improved working memory performance. Our observations are indicative of the feasibility of enhancing cognitive performance by the CFC-formed tACS.

## Introduction

Working memory is considered a cognitive system to control attention on a particular mental representation with a limited capacity that can retain information temporarily (Engle et al., 1999). Working memory has two principal mechanisms: online maintenance of information and its volitional or executive control (Miller et al., 2018). Since working memory is a fundamental feature of cognitive performance, techniques that can intentionally enhance human working memory performance would help shed light on a potent neurotechnology for human cognitive augmentations that can influence various fundamental cognitive processes. Although several studies have recently attempted to enhance the capacity of working memory (Röhner et al., 2018; Hanslmayr et al., 2019; Reinhart and Nguyen, 2019; Murphy et al., 2020), their consistency and feasibility are still contentious, and the neurophysiological rationales underlying this process are controversial.

For these reasons, neuromodulatory approaches for cognitive augmentation have been continuously updated to find reliable and robust methods consistent with their neurophysiological bases. For the practical application of neuromodulatory techniques in daily life, a non-invasive approach is favorable. For instance, transcranial current stimulation is an effective non-invasive neuromodulatory technique (Herrmann et al., 2013; Antal et al., 2017; Vosskuhl et al., 2018) including transcranial direct current stimulation (tDCS) and transcranial alternating current stimulation (tACS). Both tDCS and tACS have been shown to improve performance in memory tasks (Polanía et al., 2012; Brunoni and Vanderhasselt, 2014; Dedoncker et al., 2016; Hill et al., 2016; Mancuso et al., 2016). However, tDCS effects appear to be modest and variable between studies (Murphy et al., 2020). tACS seems to improve working memory performance more efficiently than tDCS (Röhner et al., 2018; Lang et al., 2019). It is presumed that tACS sinusoidally changes the transmembrane potential at the cellular level, an effect that is augmented through synaptically connected neurons (Ali et al., 2013). This may underlie the tACS-mediated neuromodulatory mechanism leading to entrainment and amplification of endogenous neuronal oscillations (Zaehle et al., 2010; Helfrich et al., 2014; Tavakoli and Yun, 2017; Weinrich et al., 2017; Riddle et al., 2021). These physical characteristics effectively drive the resonation of one spectral activity by others when those central frequencies are matched. Recent studies have consistently shown that tACS can be successfully used for neuromodulation in cognition and memory (Herrmann et al., 2013; Santarnecchi et al., 2013; Jausovec and Jausovec, 2014; Vosskuhl et al., 2015; Reinhart and Nguyen, 2019).

**FIGURE 1:**
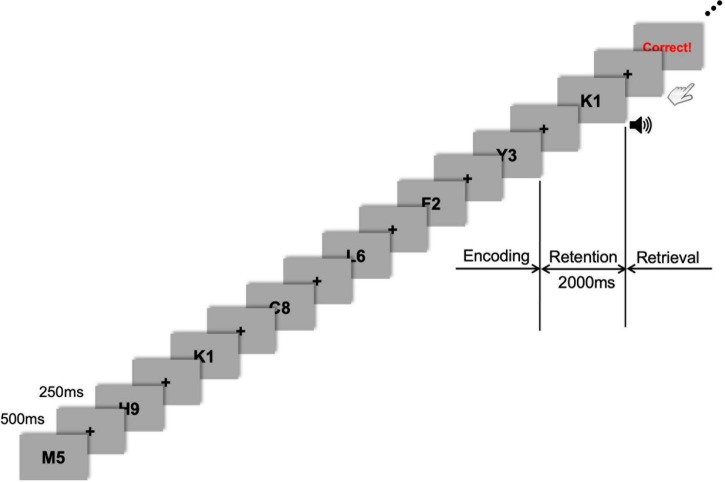
Task flow of the sample stimuli. Stimulus presentation was followed by a fixation cross presented during every inter-stimulus interval (ISI). During the encoding phase, seven letter-numeral combinations were visually presented consecutively on the display monitor for 500 ms with an ISI of 250 ms. After this encoding phase, a 2-s retention period of working memory was given before the retrieval (probe-test) phase that was cued by a 1-s auditory beep. Visual feedback (either “Correct!” or “Incorrect!” or “No Response”) was promptly shown on the monitor after each response depending on whether the probe item was part of the memory set presented during the encoding phase.

In addition, it has been reported that cross-frequency coupling (CFC) reflects a specific interplay between large ensembles of neurons, and it is likely to have profound implications for neuronal processing (Jensen and Colgin, 2007). Given that the excitability of the neurons in the network is modulated by the phase of the slow oscillations, the resulting dynamics will yield fluctuations in the fast oscillations that are phase-locked to the slow oscillations. For example, activity patterns related to multiple memories can be encoded in a single neural network that shows nested oscillations similar to those observed in the brain (Lisman and Idiart, 1995). Consistent with this model, Canolty et al. (2006) demonstrated a cross-frequency interaction between theta and gamma oscillations during working memory operations. Each memory is stored in a different high-frequency subcycle of a low-frequency oscillation. The fast oscillations divide the cycle of slow oscillations into “time slots,” which segment several active representations in time (Jensen and Colgin, 2007). Such temporal segmentation theoretically provides a principal framework responsible for the maintenance of multiple working memory items. For instance, it has been proposed that the number of gamma cycles per theta cycle determines the working memory capacity of the buffer (Jensen, 2006). The number of nested theta/gamma subcycles can account for the approximate number of items that can be held in working memory (Lisman and Idiart, 1995; Ward, 2003). Therefore, based on previous CFC-modeling studies of working memory (Lisman and Idiart, 1995; Ward, 2003; Jensen and Colgin, 2007), we hypothesized that the neuromodulatory effect of resonating the brain oscillations responsible for working memory would be maximized when the stimulus wave (i.e., tACS) resembles the target wave (i.e., human brain wave) as closely as possible. Thus, the present study employed an individually customized theta-phase/high-gamma-amplitude CFC-tACS, based on participants’ endogenous theta frequency. CFC-formed tACS has been introduced to examine its phase or amplitude dependency in cognitive control (Alekseichuk et al., 2016; de Lara et al., 2018; Akkad et al., 2019; Turi et al., 2020). In the present study, we used a peak-coupled CFC-tACS to investigate whether a peak-coupled theta-phase/high-gamma-amplitude CFC-tACS treatment perturbed the degree of ongoing CFC, possibly associated with significant changes in working memory performance. We also analyzed electroencephalographic (EEG) signals to reveal their neurophysiological signatures. For this purpose, a Sternberg task (Sternberg, 1966) with a memory-set size of seven was employed (Figure 1; see the method section for further details). Since working memory performance involves frontal theta activity (Gevins et al., 1997; Jensen and Tesche, 2002; Onton et al., 2005) and fronto-parietal theta synchronization (Sarnthein et al., 1998; Pahor and Jaušovec, 2017; Reinhart and Nguyen, 2019), and alpha activity generally reflects cortical inhibition (Klimesch, 1996; Hanslmayr et al., 2007; Mathewson et al., 2009), we hypothesized that individually customized theta peak-coupled tACS might strengthen the theta role in working memory and possibly influence its directly related or downstream processing, for instance, long-range lower frequency (e.g., delta or theta) coherence or lower-frequency-phase/high-gamma-amplitude coupling, with simultaneous suppression of inhibitory control reflected in alpha activity (leading to tACS-mediated facilitation of cortical activation) for better working memory performance. Moreover, to investigate their different behaviors in temporal neurodynamics, we analyzed EEG signals in two individual sub-intervals of the entire 2-s retention period of working memory task performance: the first half (−2,000 to −1,000 ms before the test-stimulus presentation) and second half (−1,000 to 0 ms before the test-stimulus presentation) of the retention period.

## Materials and methods

### Participants

Twenty-four healthy volunteers (nine females; mean age 23.8 years) participated in this study. Among them, 12 participants were randomly assigned to the tACS group (five females; mean age 23.5 years), and the remaining 12 participants were assigned to the sham group (four females; mean age 24.0 years). All participants had normal or corrected-to-normal vision. None reported a personal history of psychiatric or neurological disorders. All participants provided written informed consent. The study was conducted in accordance with the ethical guidelines established by the Institutional Review Board of Korea University (No. 1040548-KU-IRB-18-9-A-2).

### Materials and procedure

We used the Sternberg task to investigate the neuromodulatory effect of cross-frequency coupled tACS treatment on working memory performance (Sternberg, 1966). The Sternberg task, a classical test of working memory, is well-suited for investigating the EEG activity related to the working memory load because each trial has a well-defined retention period over which participants should hold the information of items presented in the encoding phase. It is noteworthy that both alpha and theta bands reflect neural correlates for working memory performance (Klimesch, 1999; Wianda and Ross, 2019). Since frontal midline theta activity (Gevins et al., 1997; Jensen and Tesche, 2002; Onton et al., 2005) and parieto-occipital alpha activity (Sauseng et al., 2005; Tuladhar et al., 2007; Lozano-Soldevilla et al., 2014) are associated with human performance in working memory tasks, we analyzed the frontal (i.e., Fz, FC1, and FC2), parietal (i.e., Pz, P3, and P4), and occipital (i.e., Oz, O1, and O2) EEG spectral power. We also conducted CFC, pairwise phase consistency (PPC), and nodal efficiency analyses to investigate their possible functional interplay (see below).

As a small memory-set size in the Sternberg task may lead to difficulty in disentangling the tACS effect from the sham condition, we selected a 7-item memory-span and modified a presented item (commonly either a digit or letter) into a complex one (a letter-digit combined format; Figure 1). Thus, the presented items in the Sternberg task were a combination of one letter and one digit randomly drawn from a series of single letters (A–Z) and digits (0–9). When generating sets of letter-digit stimuli for each trial, the letters and digits were randomly drawn without replacement. For example, seven letter-numeral combinations (e.g., M5, K1, Y3, and so on) were visually presented (one at a time) consecutively on the display monitor for 500 ms with an ISI of 250 ms (Figure 1). The items were presented randomly and equally often to each participant. The black-colored letter-digit combined items were presented on a gray background. The stimulus item was subtended at 5° (visual angle) and presented using presentation software (E-prime 3.0 Professional, Psychology Software Tools, Sharpsburg, USA). After this encoding phase, a 2-s retention period was given before the retrieval phase (probe-test), in which the participants had to respond by pressing a button with their right or left index finger, indicating whether they believed the probe item (i.e., test-stimulus) either was or was not part of the memory set presented during the encoding phase. The task probability whether the probe item was or was not part of the memory set was 50–50. Response hands were counterbalanced across participants. A 1-s auditory cue (beep sound) was presented just after the 2-s retention phase to prepare participants for the upcoming probe-test and consequent response. The probe was presented until the participant responded or 5 s elapsed, which was the maximum response window. The participants were required to press the button as quickly as possible. Following their response, visual feedback (either “Correct!” or “Incorrect!” or “No Response”) was promptly shown on the monitor to motivate participants’ better task performance. The inter-trial interval was 1 s, which began with the probe offset and ended with the onset of the next trial. Each participant performed 60 trials. This Sternberg task was repeatedly applied to the same participant after either 20 min of tACS or sham stimulation to investigate the effect of tACS treatment on working memory performance. Participants were debriefed immediately after the last session. During the debriefing, participants indicated their subjective perception (or any uncomfortable experience, including retinal phosphenes) of stimulation.

### Transcranial alternating current stimulation and electroencephalographic recordings

**FIGURE 2:**
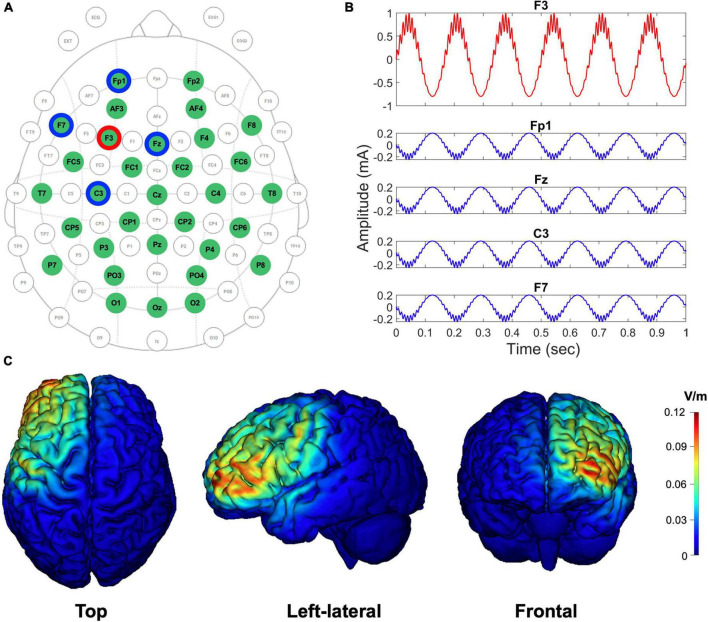
EEG-tACS 32-channel configuration, the shape of the CFC-tACS stimulus, and tACS cortical target areas by simulation. **(A)** EEG and tACS channel montage. The red ring indicates a stimulation channel of F3, and its four surrounding return channels (i.e., Fp1, Fz, F7, and C3) are highlighted in blue rings. Using the remaining channels in the green circles, the EEG signals were recorded. The topographical view is from the vertex, with the nose at the top of the image. **(B)** The shapes of theta-phase/high-gamma-amplitude coupled tACS stimulus (1.8 mA peak-to-peak). The form of an input CFC-tACS stimulus (red curve) is illustrated in F3, and the sum of the remaining four return signals (blue curves) has the same amplitude as the F3 input signal, but with its reverse polarity and in anti-phase to disperse the return current effects. **(C)** The cortical-level tACS target (i.e., left prefrontal) areas are simulated using the scalp-level tACS electrode configuration. Top, left-lateral, and frontal views of cortical maps are illustrated from left to right. Color scales indicate the intracranial electric field (V/m), with high intensity in red and low intensity in blue.

Transcranial alternating current stimulation was delivered between the two Sternberg task performances using a Starstim R32 Neurostimulator (Neuroelectrics, Barcelona, Spain) at an intensity of 1.8 mA peak-to-peak. Since the left prefrontal area has been considered essential for working memory function (Gabrieli et al., 1998; Mull and Seyal, 2001; Fregni et al., 2005), electrode F3 was selected as the stimulation channel, and a set of return electrodes (Fp1, Fz, F7, and C3) were arranged in a ring around the central stimulation electrode (Figure 2A). The currents of all stimulation and return channels were set to keep the total amount of current at a zero sum. The tAC stimulus wave was designed as a cross-frequency (theta-phase and high-gamma-amplitude) coupled pattern (Figure 2B) using the following equations coded in MATLAB (ver. R2019a, MathWorks, Natick, USA):


$$S\,t\,i\,m\,u\,l\,u\,s\,s\,i\,g\,n\,a\,l=\frac{{\bar{A}}_{f_{A}}}{2}\,\left(\sin\,\left(2\,\pi \,f_{P}\,t\right)+1\right)\,\sin\left(2\,\pi \,f_{A}\,t\right)+{\bar{A}}_{f_{p}}\,\sin\left(2\,\pi \,f_{P}\,t\right)$$

$$R\,e\,t\,u\,r\,n\,s\,i\,g\,n\,a\,l=-\frac{1}{4}\,\left(S\,t\,i\,m\,u\,l\,u\,s\,s\,i\,g\,n\,a\,l\right)$$

where *f_A_* and *f_P_* are amplitude and phase frequency, respectively. ${\bar{A}}_{f_{A}}$ and ${\bar{A}}_{f_{P}}$ are constants that determine the maximum amplitude of *f_A_* and *f_P_*, respectively.

CFC-tACS stimulus was generated with a sampling frequency of 1,000 Hz. ${\bar{A}}_{f_{A}}$ and ${\bar{A}}_{f_{P}}$ were set to 0.2 and 0.8, respectively. The theta frequency (*f_P_*) for the generation of the stimulus was individually selected, where dominant theta activity was observed based on the fast Fourier transformed theta-band (3–8 Hz) results on EEG during the retention period of task performance before stimulation. When the peak frequency was not clearly detected within the theta band, the frequency exhibiting global maximum power within the theta frequency range was selected. The gamma frequency (*f_A_*) for the CFC-tACS stimulus was calculated by multiplying the individual theta frequency by 16 because we hypothesized that the gamma frequency has an integer-fold relationship to the theta frequency based on the CFC-memory model (Lisman and Idiart, 1995; Ward, 2003) and we confined the high-gamma range to 50–100 Hz. The multiplication number of 16 was chosen because the center frequency of the theta band was assumed as 5 Hz and the efficient gamma frequency of CFC-tACS for working memory performance was approximately 80 Hz (i.e. 16 × 5 = 80) in a previous study (Alekseichuk et al., 2016). It has been reported that gamma power correlates with the number of objects held in working memory (Howard et al., 2003; Roux et al., 2012) and that the number of gamma cycles per theta cycle determines the working memory capacity of the buffer (Jensen, 2006). To use the individually customized CFC waveforms in tAC stimulation protocols, the generated CFC-formed tAC stimulus file was loaded into a Starstim R32 Neurostimulator. The stimulation duration was 20 min with a 10-s ramp-up and ramp-down for each stimulus. The sham group was treated only with a 10-s ramp-up and ramp-down for each stimulus during the 20-min stimulation duration, but not with in-between tACS stimulation. During the stimulation, participants were instructed to look at a fixation cross on the monitor under dim light. Using an oscilloscope, it was double-checked whether the CFC-formed signals physically generated the exact amplitude, shape, and frequency that were intended during the design phase. Using tES LAB software (ver. 3.0.6.2, Neurophet, Seoul, South Korea), simulated cortical maps of the electric field distribution were computed using our tACS-stimulation parameters to check whether the stimulation site was optimally placed on the position that we intended to stimulate. NG Pistim electrode (Neuroelectrics, Barcelona, Spain) is an electrode that can be used for EEG monitoring and/or transcranial electrical stimulation. NG Pistim is an Ag/AgCl electrode with a radius of 1 cm. Owing to its π cm^2^ contact area, Pistim is ideal for multielectrode stimulation protocols. A neoprene headcap and gel-based electrodes were used for both EEG recording and tACS stimulation. The electric field intensities (V/m) of cortices targeted using a set of stimulation and return channels were estimated, and the results were color-coded in the cortical maps (Figure 2C). The electric field with an intensity of approximately 0.12 V/m at the activated cortical region has been reported to induce effective tCS-mediated neuromodulation in previous studies (Cancelli et al., 2016; Dmochowski et al., 2017; Kar et al., 2017). During the Sternberg task performance, EEG signals were measured using the same Starstim device at a sampling rate of 500 Hz during recording with 32 electrodes in accordance with the international 10–20 system. The online reference electrode, which was designed as the reference electrode in a Starstim R32 Neurostimulator, was placed on the right earlobe. Electrode input impedances were maintained below 10 kΩ prior to data acquisition. No online filter was applied during EEG acquisition (neither bandpass nor notch filter).

### Data analysis

Reaction times and the accuracy of task performance were measured for behavioral analysis. Only trials with correct responses were collected for further analysis. Reaction times were collected within their individual 95% confidence intervals. To control individual variances in behavioral data, the post-treatment values in both reaction times and accuracies were individually normalized by their pre-treatment values.

During offline pre-processing, a 0.5-Hz high pass filter and 60-Hz notch filter were applied to the raw EEG signals. The EEG signals with an average reference were segmented from 2,500 ms pre-stimulus to 500 ms post-stimulus for each trial. For each EEG epoch, the segment was analyzed in the time window from the test-stimulus onset to 2 s before the test-stimulus presentation (i.e., a retention period). EEG epochs with amplitudes greater than +100 μV or less than −100 μV and included a gradient greater than 50 μV/ms were automatically excluded. In addition, EEG epochs contaminated by eye movement were rejected by manual inspection. The artifact rejection rate of the EEG epochs was 3.7% on average. Two participants (one for each group) were excluded from further analyses due to poor data quality.

The power of oscillatory activity was investigated by convolving the EEG signals with Morlet wavelets (Herrmann et al., 2005). The Morlet-convolved signal shows a Gaussian envelope with a temporal standard deviation (σ_*t*_) and a spectral standard deviation [σ_*f*_ = 1/(2πσ_*t*_)] around its central frequency (*f_0_*).


$$\psi \,\left(t,f\right)=A\,e^{i\,2\,\pi \,f\,t}\,e^{\left(\frac{-t^{2}}{2\,\sigma_{t}^{2}}\right)}$$

To have unit energy at all scales, the wavelet functions should be normalized prior to the convolution. For the Morlet wavelet transform, the normalization parameter *A* is $\sigma_{t}^{-1/2}\,\pi^{-1/4}$. A wavelet family is characterized by a constant ratio (*f*_0_/σ_*f*_), and we employed a wavelet family with 7 as its constant ratio (Tallon-Baudry et al., 1997) and *f_0_* ranging from 3 to 13 Hz in 0.5 Hz steps. In the case of 10 Hz, this yields a wavelet duration (2σ_*t*_) of 222.8 ms and a spectral bandwidth (2σ_*f*_) of 2.9 Hz around its central frequency (*f_0_* = 10 Hz). The wavelet transform was conducted for each individual trial, and the absolute values of the resulting transforms were averaged. This measure of signal amplitude in single trials reflects the total activity for a certain frequency range. Because the brain oscillations in the mixed alpha and theta bands have been determined to be the most dominant brain activity during the retention phase of working memory task performance (Klimesch, 1999; Onton et al., 2005; Raghavachari et al., 2006; Meltzer et al., 2008; Wianda and Ross, 2019), we investigated whether alpha and theta power reflect neural correlates for working memory performance during the retention mental state across the tACS-treated and sham groups. We confined the alpha activity to the frequency range of 8–13 Hz and the theta activity to the frequency range of 3–8 Hz. The individual alpha or theta frequencies were determined individually for every participant, as the dominant peak frequency within each alpha or theta frequency band varied between participants.

Since early and late stages of working-memory maintenance contribute differentially to memory formation (Bergmann et al., 2013), to evaluate the tACS effect in the first half and second half of the retention period individually, we measured the mean power of alpha or theta activity in two different time windows: a first half from 2,000 to 1,000 ms before the test-stimulus presentation and a second half from 1,000 to 0 ms before the test-stimulus presentation. No baseline correction was applied to the total power, as alpha activity in the pre-stimulus period vanishes after baseline correction.

Based on the areas of the brain where the EEG oscillatory activity was most pronounced during the Sternberg task, three frontal channels (i.e., Fz, FC1, and FC2), three parietal channels (i.e., Pz, P3, and P4), and three occipital channels (i.e., Oz, O1, and O2) were selected for time-frequency analysis (Supplementary Figure 1). The average *Z*-scores of subtractions (post-stimulus spectral power minus pre-stimulus spectral power) across the selected electrodes were analyzed at their dominant peaks within each corresponding time window. *Z*-score normalization refers to the process of normalizing every post-pre-subtraction value of each electrode in a dataset such that the mean of all the post-pre-subtraction values over all electrodes is 0 and its standard deviation is 1. The measures were analyzed using independent-sample Mann-Whitney *U* tests (for a between-subjects design) and Wilcoxon signed-rank tests (for a within-subject design). All analyses were performed using MATLAB including a wavelet toolbox (ver. R2019a, MathWorks, Natick, USA) or SPSS Statistics (ver. 25, IBM, Armonk, NY, USA).

### Cross-frequency coupling analysis

To investigate the CFC, we computed the coherence between the low-frequency (1–13 Hz; less than the beta band) signal and time course of power at higher frequencies (up to 100 Hz) using Brainstorm software (Tadel et al., 2011). Coherency *Coh(f_1_,f_2_)* was estimated between the signal *X*_*t*_ and the estimated time course of power *P_*t*_(f_2_)* for a given frequency *f*_1_ by applying a sliding Hanning tapered time window followed by Fourier transformation. We applied a Hanning taper to an adaptive time window of six cycles for each frequency (ΔT = 6/f). Coherency was calculated with respect to the two time-series, as shown in the following equation:


$$\mathrm{Coh}\,\left(f_{1},f_{2}\right)=\frac{\sum_{k=1}^{M}{\tilde{X}}^{k}\,\left(f_{1}\right)\,{\tilde{P}}^{*k}\,\left(f_{1},f_{2}\right)}{\sqrt{\sum_{k=1}^{M}{\left|{\tilde{X}}^{k}\,\left(f_{1}\right)\right|}^{2}\,\sum_{k=1}^{M}{\left|{\tilde{P}}^{k}\,\left(f_{1},f_{2}\right)\right|}^{2}}}$$

where


$${\tilde{X}}^{k}\,\left(f_{1}\right)=△\,\text{t}\,\sum_{l=1}^{M}h_{l}\,X_{L\,\left(k-\frac{1}{2}\right)+l}\,e^{-i\,2\,\pi \,f_{1}\,l\,△\,t}$$

$${\tilde{P}}^{k}\,\left(f_{1},f_{2}\right)=△\,\text{t}\,\sum_{l=1}^{M}h_{l}\,P_{L\,\left(k-\frac{1}{2}\right)+l}\,\left(f_{2}\right)\,e^{-i\,2\,\pi \,f_{1}\,l\,△\,t}$$

Hence, Δ*t* = 1/*F*_*s*_, where *F*_*s*_ is the sampling frequency. The length of the time window decreased with the frequency: *K = F_*s*_M/f_2_*, where *M* is the number of cycles per time window. Coherency was computed with respect to the two time-series divided into *M* segments, each with 2,048 data points in length (*L*) (Osipova et al., 2008). Coherence is the absolute value of coherency |*Coh(f_1_,f_2_)*|. In this case, *h*_*l*_ in the above equation set refers to a 2,048-point Hanning window, and * refers to the complex conjugate. This allowed us to perform a sensor-by-sensor characterization of phase-to-power cross-frequency interactions with respect to *f*_1_ and *f*_2_. To compute spectrograms of EEG activity averaged at the phase troughs of the low frequency with the highest phase-amplitude coupling (PAC), the signals were first filtered at the delta band using a narrow band-pass filter. The amplitude troughs of the delta frequency were then detected from the filtered signal. A time window from −1 to 1 s was extracted relative to these marked troughs, and a time-frequency decomposition of these epochs was performed to obtain the power of all the averaged time-frequency plots using the Brainstorm software (Tadel et al., 2011).

Since frontal top-down processing controls bottom-up visual information from occipital cortices (Barcelo et al., 2000), the two representative electrodes Fz and Oz were selected for the CFC analysis. Reciprocal connections between the frontal and the occipital cortices through the fronto-occipital fasciculi (Wakana et al., 2004) provides a neuroanatomical substrate for control. Given that the prefrontal region (representative by electrode Fz) is associated with memory retrieval (Tomita et al., 1999; Lepage et al., 2000) and top-down processing (Min and Park, 2010; Keeser et al., 2011), and that the occipital region (representative by electrode Oz) is related to memory-encoding (Makovski and Lavidor, 2014; Yin et al., 2020) and visual bottom-up processing (Ro et al., 2003; Ekstrom et al., 2008), we examined bi-directional Fz-phase/Oz-amplitude and Oz-phase/Fz-amplitude CFCs. The CFC value of the stimulus-induced differences (post-stimulus CFC minus pre-stimulus CFC) were compared between tACS-treated and sham groups using a cluster-based permutation test to investigate the degree of tACS-mediated changes in CFCs. Statistical inference for random effects analysis relied on non-parametric permutation tests because they make minimal assumptions on the distribution of the data. To detect significant cross-frequency modulation in a phase-amplitude coupling map, we used cluster-size permutation tests (Maris and Oostenveld, 2007) with *p* < 0.05 cluster defining threshold and *p* < 0.05 cluster threshold. The permutation tests were one-sided with independent (unpaired) samples. We computed 5,000 permutation samples (including the original data) to estimate the data null distribution.

### Functional connectivity analysis

A network represents a mathematical model in which a complex system can be decomposed into elements (i.e., nodes) and their interactions (i.e., links or connections) (Kim and Min, 2020). The association between these elements in the brain network is generally described by its structural or functional connectivity. Anatomical connections between brain regions provide the structural basis for functional connectivity, which assesses the statistical dependence of neural signals across spatially remote brain regions. Synchronization of neuronal oscillatory activity among functionally associated and widely distributed brain areas has been recognized as a principal mechanism in the integration of brain signals underlying cognitive processes (Hummel and Gerloff, 2005; Hipp et al., 2011). Among several approaches that infer functional connectivity of inter-regional brain oscillations, phase synchronization measures are conventionally used (Varela et al., 2001; Van Diessen et al., 2015). The most widely used phase interaction metric is the phase-locking value (PLV), a measure of the phase synchrony between two time-series (Lachaux et al., 1999; Mormann et al., 2000). The PLV ranges from 0 to 1, with 0 representing perfect non-phase-locking and 1 indicating 100% phase-locking. However, the PLV has biased estimators for finite sample sizes and is thus vulnerable to the number of observations.

As an alternative measure of phase consistency, PPC has been proposed (Vinck et al., 2010). Unlike the PLV, the PPC quantifies the distribution of all pairwise differences between the relative phases. The distribution of pairwise phase differences is largely concentrated around an average value in the existence of phase synchronization. As shown in the following equation, the PPC computes the cosine of the angular distance for all pairs of relative phases. Therefore, it represents how similar the relative phase of one trial is to the relative phase of another trial. Since the PPC is based on sequential pairs of observations, it mainly considers the consistency of phase differences and does not suffer from bias due to a finite sample size. Thus, the advantage of PPC over PLV is that this metric is consistent and unbiased by a finite number of sample sizes, such as in EEG studies, wherein the number of artifact-free trials is typically not sufficient and varies across participants. Therefore, we analyzed the PPC in the present study. To compute the PPC, we used the FieldTrip toolbox (Oostenveld et al., 2011). The PPC was computed as follows:


$$P\,P\,C=\frac{2}{N\,\left(N-1\right)}\,\sum_{i=1}^{N-1}\sum_{j=\left(i+1\right)}^{N}\cos\left(\theta_{i}-\theta_{j}\right),$$

where *N* is the number of trials, and θ_*i*_ and θ_*j*_ represent the relative phases of trials *i* and *j*, respectively. The cosine was computed between all trial-pairwise combinations of relative phases, and the PPC was taken as the mean of these cosine values. PPC values range between −1 and 1, with positive values indicating phase synchronization and zero for random phases. To compute the phases of the brain oscillations, we performed a discrete Fourier transformation on the EEG signals with a 1-s Hanning window. We used a common average reference (CAR) to mitigate the effect of volume conduction (Ludwig et al., 2009; Tsuchimoto et al., 2021). PPC analysis was performed across the frontal (Fz, F3, F4, FC1, and FC2) and parieto-occipital (Pz, P3, P4, Oz, O1, and O2) regions. To statistically compare PPC values between pre- and post-treatment, Wilcoxon signed-rank tests were used. A false discovery rate (FDR) of *q* < 0.05 (Benjamini and Hochberg, 1995) was used to correct for multiple comparisons.

### Functional network analysis

Topological properties of brain networks offer the possibility of characterizing whole-brain organization. The functional network is substantiated in terms of the statistical correlation between signals recorded from each brain region and measured using various approaches. We performed a graph-theoretical analysis (Bullmore and Sporns, 2009; Khan et al., 2018) to assess the network properties (nodal efficiency) using the brain connectivity toolbox (Rubinov and Sporns, 2010) and EEGNET (Hassan et al., 2015). Graph theory provides a variety of tools for analyzing complex brain networks and characterizing the organization of the brain (Kim and Min, 2020). Network integration indicates the ability to combine information of various brain areas and transmit information in the network.

To compute the local network attribute for integrating information, we computed the nodal efficiency using the following equation:


$$E^{n\,o\,d\,a\,l}\,\left(i\right)=\frac{1}{N-1}\,\sum_{j\neq i}\frac{1}{d_{i\,j}}$$

where *N* is the number of nodes composing the graph, and *d*_*ij*_ is the shortest path length between node *i* and node *j*. Nodal efficiency measures the ability of information propagation for a specific node.

As multiple comparisons across all channels can cause type-I errors, the cluster-based permutation test method was used to statistically analyze the nodal efficiency (Maris and Oostenveld, 2007). This method was conducted by randomly permuting the nodal efficiencies between the pre- and post-treatment within each participant. By creating a reference distribution from 5,000 random sets, the *p*-value was estimated as the proportion of the elements in the randomization null distribution exceeding the observed maximum cluster-level test statistic. Each cluster was formed based on spatial adjacency, where channels were considered neighbors if the distance between them was less than 6 cm. The distance of 6 cm was chosen based on the common distance between adjacent scalp electrodes. In addition, the threshold for estimating the *p*-value was the 2.5th or 97.5th quantile, which was used as a critical value at an alpha-level of 0.05. A FDR of *q* < 0.05 (Benjamini and Hochberg, 1995) was used to correct for multiple comparisons.

## Results

**FIGURE 3:**
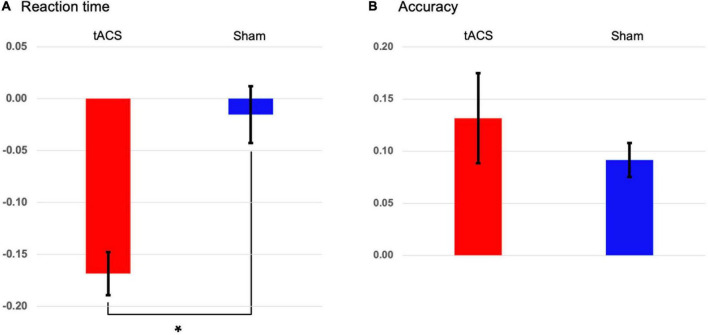
Transcranial alternating current stimulation (tACS)-mediated changes in the behavioral data. The normalized changes between pre- and post-treatment (post-treatment minus pre-treatment) in **(A)** reaction times and **(B)** accuracies of task performance in both the tACS-treated (red bars) and sham (blue bars) groups. The error bars indicate the standard errors of the mean, and the asterisk represents statistical significance (*p* < 0.001).

None of the participants indicated stimulation-related scalp sensations/adverse reactions or phosphenes during stimulation. During the retrieval (test) phase of the Sternberg task, 24 participants were instructed to report whether the probe item was part of the memory set presented during the encoding phase by pressing a button. We found that tACS treatment yielded a significant reduction of individually normalized differences in reaction times between pre- and post-treatment (*p* < 0.001: tACS-treated, −0.169; sham, −0.015, Figure 3A). This finding shows that tACS treatment enhanced working memory performance. Although individually normalized differences in the accuracy of task performance between pre- and post-treatment of the tACS-treated group (0.132) tended to be higher than those of the sham group (0.092), they did not reach statistical significance (Figure 3B).

**FIGURE 4:**
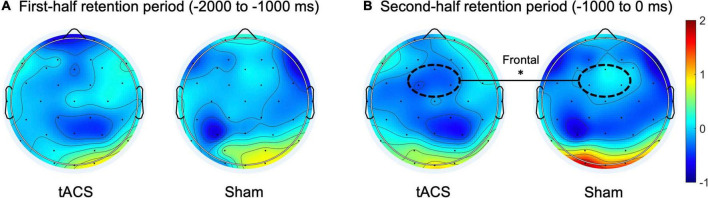
Transcranial alternating current stimulation (tACS)-mediated topographical changes in alpha power. The grand-averaged *Z*-scores of differences in alpha power (normalized *Z*-scores at individual alpha frequencies) between pre- and post-treatment (post-treatment minus pre-treatment) during **(A)** the first half and **(B)** the second half of the retention periods in both the tACS-treated and sham groups. The dotted curves indicate the frontal (Fz, FC1, and FC2) region of interest. The asterisk represents statistical significance between tACS-treated and sham groups (*p < 0.01). The view of the topography is from the vertex, with the nose at the top of the image.

Regarding the normalized differences (i.e., *Z*-scores) in EEG alpha and theta power between pre- and post-treatment, during the second half of the retention period (−1,000 to 0 ms before the test-stimulus presentation), the tACS-treated group showed significantly reduced alpha power as compared to the sham group in the frontal region (*p* < 0.01: tACS-treated, −0.359 μV^2^; sham, −0.017 μV^2^; Figure 4). The other experimental conditions did not show significant differences in EEG spectral power.

**FIGURE 5:**
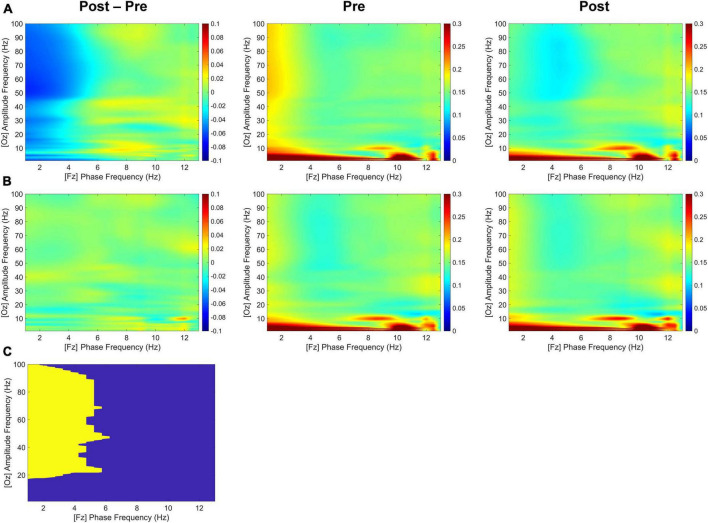
Transcranial alternating current stimulation (tACS)-mediated cross-frequency phase-amplitude couplings. The grand-averaged differences in phase-amplitude couplings between pre- and post-treatment (post-treatment minus pre-treatment) as a function of analytic amplitude (1−100 Hz) and analytic phase (1−13 Hz) for the Fz-phase/Oz-amplitude combination during the second half (−1,000 to 0 ms before the test-stimulus presentation) of the retention periods in both **(A)** the tACS-treated and **(B)** the sham groups. Their pre- and post-treatment conditions are also individually displayed. **(C)** The statistically significant regions are highlighted in yellow [one-sided permutation test with independent (unpaired) samples, *p* < 0.05 cluster defining threshold; *p* < 0.05 cluster threshold]. The color bar indicates differences in coherence between pre- and post-treatment.

As shown in Figure 5, although both delta and theta bands exhibited noticeable phase modulations, the delta-phase/high-gamma-amplitude coupling was broadband up to 100 Hz, with the strongest modulation occurring from 20 to 100 Hz. During the second half of the retention period, the tACS-treated group showed significantly greater mean differences in CFC values within 1–4 Hz phase frequency and 20–100 Hz amplitude frequency after minus before treatment than the sham group for the Fz-phase/Oz-amplitude coupling (*p* < 0.05; tACS-treated, −0.048; sham, −0.001). *Post hoc* tests revealed that the CFC values of the tACS group were significantly lower than those of the sham group in the post-treatment (*p* < 0.05; tACS-treated, 0.128; sham, 0.155). However, there were no significant differences in the Oz-phase/Fz-amplitude coupling during the first half (−2,000 to −1,000 ms before the test-stimulus presentation) and the second half (−1,000 to 0 ms before the test-stimulus presentation) of the retention periods. These observations are indicative of unidirectional modulation from frontal top-down areas to occipital bottom-up areas.

**FIGURE 6:**
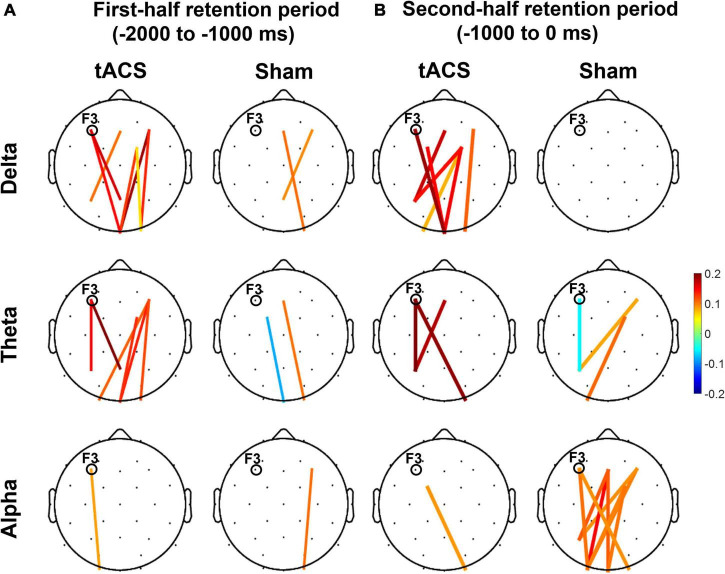
Topographical changes in pairwise phase consistency (PPC) between pre- and post-treatment in the delta, theta, and alpha bands. The color-coded lines in the grand-averaged topographies represent differences in PPC between pre- and post-treatment (post-treatment minus pre-treatment) during **(A)** the first half (−2,000 to −1,000 ms before the test-stimulus presentation) and **(B)** the second half (−1,000 to 0 ms before the test-stimulus presentation) of the retention periods in both the tACS-treated and sham groups. The red lines indicate a significant enhancement of PPC connection strength, whereas the blue lines indicate a significant reduction of PPC connection strength. Only the statistically significant differences in PPC connections (*p* < 0.05, FDR-corrected) between the frontal (Fz, F3, F4, FC1, and FC2) and parieto-occipital region (Pz, P3, P4, Oz, O1, and O2) are displayed in colored lines. The electrode position for tAC stimulation (i.e., F3) is marked in each topography. The view of the topography is from the vertex, with the nose at the top of the image.

During the second half of the retention period of working memory, the sham group showed greater differences in occipital alpha power between pre- and post-treatment (post-treatment minus pre-treatment) and pronounced functional connectivity in the alpha band among the frontal, parietal, and occipital areas (Figure 6). In contrast, there was a clear absence of significant F3-centered functional connectivity in the alpha band during the second half of the retention period in the tACS-treated group. The tACS-treated group also showed significant enhancement of functional connectivity in lower frequencies such as the delta and theta bands over the frontal, parietal, and occipital areas.

**FIGURE 7:**
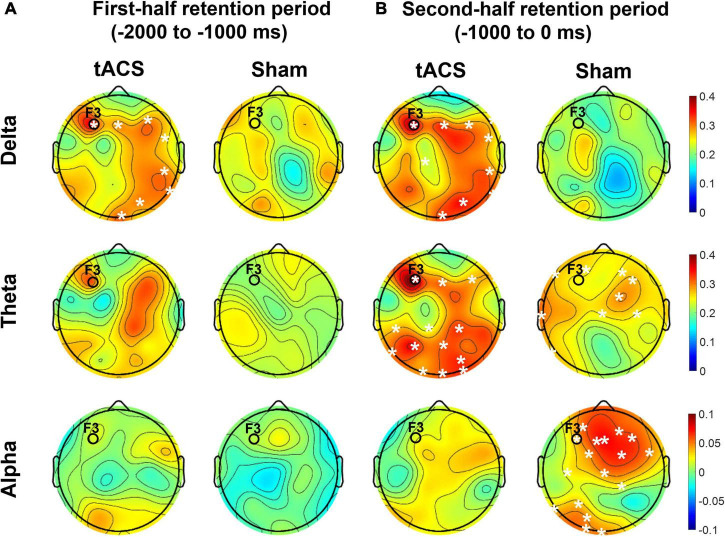
Topographical distributions of the differences in nodal efficiency between pre- and post-treatment in the delta, theta, and alpha bands. The grand-averaged topographies represent the differences in nodal efficiency between pre- and post-treatment (post-treatment minus pre-treatment) during **(A)** the first half (−2,000 to −1,000 ms before the test-stimulus presentation) and **(B)** the second half (−1,000 to 0 ms before the test-stimulus presentation) of the retention periods in both the tACS-treated and sham groups. The asterisks represent statistical significance (*p* < 0.05, FDR-corrected). The electrode position for tAC stimulation (i.e., F3) is marked in each topography. The view of the topography is from the vertex, with the nose at the top of the image.

As shown in Figure 7, the differences in nodal efficiencies between pre- and post-treatment (post-treatment minus pre-treatment) were significantly enhanced over the frontal, parietal, and occipital areas in the tACS group. During the second half retention period of the sham group, the differences in the nodal efficiency of theta activity between pre- and post-treatment increased only in the frontocentral region but not in the occipital region. It is noteworthy that strong nodal efficiency of alpha activity in the sham group was suppressed in the tACS-treated group during the second half of the retention period (Figure 7).

## Discussion

We observed a significant improvement in working memory performance using the individually customized CFC-formed tACS treatment. In addition to the significant reduction in reaction times, several analyses of EEG signals also substantiated the effect of tACS on working memory performance. Based on the tACS-mediated suppression in the frontal alpha power during the second half of the retention period, tACS might play a promotive role in facilitating the sustained maintenance of encoded information at the later stage of the retention phase for better performance of working memory. Although the present CFC-tACS did not include a spectral component of alpha frequency, the present suppression of frontal alpha activity may reflect a downstream effect of CFC-tACS, possibly leading to changes in visual awareness and reaction time consequently. This is because synchronous oscillations in the alpha band are generally indicative of a state of cortical inhibition that affects visual awareness (Klimesch, 1996; Hanslmayr et al., 2007; Mathewson et al., 2009), and a large event-related desynchronization of alpha power is thus associated with good memory performance (Klimesch et al., 1997a,^2006^; Klimesch, 1999; Doppelmayr et al., 2005). Consistently, the sham group showed pronounced alpha power during the retention period, resulting in poor memory performance compared to the tACS-treated group. In other words, alpha suppression reflects an increased excitability level of neurons in the involved cortical areas, which may be related to enhanced information transfer in the corresponding thalamocortical circuits (Neuper and Pfurtscheller, 2001). Since the increase in alpha desynchronization reflects an increase in general attentional demands (Klimesch et al., 1997b,^2007^), the alpha suppression during the retention period may indicate tACS-mediated enhancement of attentional processes for better performance of working memory.

Furthermore, directional delta/high-gamma coupling in tACS-treated participants (i.e., stronger in the Fz-phase and Oz-amplitude CFC, but weaker in the Oz-phase and Fz-amplitude CFC) was suggestive of unidirectional modulation from frontal top-down areas to occipital bottom-up areas. As high-gamma (> 50 Hz) CFC is important for understanding effective local and inter-regional communication during cognitive processing in humans (Canolty et al., 2006; Canolty and Knight, 2010), the present study provides evidence of the frontal top-down modulation of working memory. Particularly during the second half of the retention period of the tACS-treated group, CFC analyses revealed a directional inhibitory control on the occipital area by the frontal area. Presumably, the first half of the retention period would serve as a precedence duration for a ramp-up effect, resulting in the lack of significant CFC-tACS effects. Rather, during its consecutive second half retention period, the F3-targeted tACS might effectively regulate occipital CFC networking preparing the upcoming retrieval period, leading to the improvement of working memory performance. In accordance with previous frontal alpha suppression by tACS treatment, these observations indicate that tACS might engage in frontal top-down regulation to the early stage of visual processing in the occipital area. Given that the prefrontal cortex is essential for goal-directed behaviors and higher-order cognitive control (Miller and Cohen, 2001; Fuster, 2013) and CFC-formed tACS modulated task performance of the associated cognitive control component (Riddle et al., 2021), our observations suggest that the CFC-formed tACS treatment plays an assistive role to the frontal working memory function for improving its performance. Presumably, during the retention phase, the visual aspects of encoded information around the visual cortices might be further rehearsed for better working memory performance, which could be accomplished by tACS-mediated top-down control of the frontal area. Although the present study examined only the frontal-occipital bidirectional influence using PAC analysis across electrodes Fz and Oz, lateralized influence can additionally be investigated using lateralized electrodes in the anterior and posterior regions in future studies.

These observations are further supported by the nodal efficiency and PPC results. As shown in Figure 6, during the second half of the retention period, the sham group exhibited robust F3-centered functional connectivity in the alpha band among the frontal, parietal, and occipital areas, whereas the tACS-treated group showed a lack of F3-centered functional connectivity in the alpha band. Since tACS was delivered to the frontal area (i.e., F3), these F3-centered brain connectivity changes might reflect the effect of tACS treatment. Consistently, as shown in Figure 7, strong nodal efficiency of alpha activity in the sham group was suppressed in the tACS-treated group during the second half of the retention period. Nodal efficiency measures the ability of information propagation between a node and the remaining nodes in the network (Achard and Bullmore, 2007). Since alpha activity generally reflects cortical inhibition (Klimesch, 1996; Hanslmayr et al., 2007; Mathewson et al., 2009), these observations of disinhibition in the alpha band are all indicative of tACS-mediated facilitation of cortical activation for better working memory performance.

In addition, the tACS-treated group exhibited enhancement of F3-centered functional connectivity in lower frequencies such as the delta and theta bands during the second half of the retention period when tACS-mediated suppression of alpha power and the absence of F3-centered functional connectivity were detected (Figure 6). These observations are consistent with the topographical distributions of their nodal efficiencies (Figure 7). In the tACS group, the nodal efficiency of the delta and theta bands was consistently enhanced in the frontal, parietal, and occipital areas. These findings support that the CFC-tACS treatment promotes the enhancement of inter-regional information flow related to executive function and memory storage, resulting in better working memory performance.

It has been consistently reported that working memory performance was revived in older adults by the improvement of long-range theta synchronization (Reinhart and Nguyen, 2019) and that working memory involves theta coherence in the fronto-parietal network (Sarnthein et al., 1998; Pahor and Jaušovec, 2017). These frequency-specific communications in the fronto-parietal network have been consistently reported in previous studies, showing that the fronto-parietal coupling in the theta band differentiated between memorized items with the involvement of information transmission (Jacob et al., 2018), and that tACS increases global phase connectivity in a strong association with improvement of spatial working memory performance (Alekseichuk et al., 2016). Consistently, as frontal theta networks coordinate inter-regional communication (Voytek et al., 2015), tACS might engage in network communications for better working memory performance. As shown in Figure 6, the frontal-parietal-occipital connection of the tACS group was notably strengthened, and this result is consistent with previous studies on distributed fronto-parieto-occipital processing stages during working memory (Halgren et al., 2002; Yu and Shim, 2017; Yu et al., 2020) and the parieto-frontal integration theory (P-FIT) (Jung and Haier, 2007), in which human intelligence is interpreted in terms of interactions within the frontal and parietal cortical regions. For example, the central executive function of working memory is involved in the frontal area, whereas the working memory storage component is associated with the parietal area (Sauseng et al., 2005, 2009, 2010). Consistently, human neuroimaging studies have revealed that information held in visual working memory is represented in the frontal (Lee et al., 2013), parietal (Bettencourt and Xu, 2016), and occipital cortices (Harrison and Tong, 2009). As found in the present study, event-related and coherent phase-locked oscillations supported functional interaction and these dorsal stream structures throughout the task performance. These results indicate how a fronto-parietal “central executive function” might interact with an occipital “visuospatial sketch pad function.”

Taken together, our observations indicate neurophysiological correlates of tACS-mediated changes during working memory performance. Such prominent frequency-dependent neurodynamics of nodal efficiency between the frontal and occipital regions reinforce the previous unidirectional Fz/Oz CFC observations in the tACS-treated group. Therefore, these observations provide promising evidence that CFC-formed tACS treatment effectively influences the electrophysiological changes that underlie a significant improvement in working memory performance. Thus, theta-phase/high-gamma-amplitude coupling tACS can be a potent tool to augment human cognitive functions such as working memory. Nevertheless, the present study has several limitations. First, although a larger sample size would have improved the statistical power of our study, sample sizes were limited by the tACS treatment/sham subgrouping. Despite the use of nonparametric statistical tests (e.g., Mann-Whitney *U* test), the limited statistical power should be carefully considered when interpreting our observations. Second, we investigated the tACS effect only during the retention period. Since a working memory task consists of encoding, retention, and retrieval phases, further detailed experimental designs are necessary to examine the effect of tACS on working memory performance individually on each encoding, retention, or retrieval phase. Third, multiple targets of neuromodulation can also be proposed to better investigate the effect of tACS on working memory. Furthermore, although there are currently technical limitations, it is necessary to selectively target and expose tACS efficiently and selectively to the deep brain (subcortical) structures such as the hippocampus (Mitchell et al., 2000; Axmacher et al., 2010) or medial prefrontal cortex (Keller et al., 2015; Müller et al., 2020), which are crucial for working memory function. Fourth, the manipulation of phases (either in-phase or anti-phase) of tACS waveforms on multiple targets could also be manipulated to find an optimal tACS technique for effective neuromodulation. All of these points will be considered in future research with an advanced experimental design.

## Data availability statement

The data has not been shared in a public repository due to the privacy rights of human subjects. However, the data and analysis tools used in the current study are available from the corresponding author upon reasonable request.

## Ethics statement

The studies involving human participants were reviewed and approved by the Institutional Review Board of Korea University (No. 1040548-KU-IRB-18-9-A-2). The patients/participants provided their written informed consent to participate in this study.

## Author contributions

B-KM conceived and designed the CFC-tACS paradigm to investigate its effect on working memory performance and performed research. B-KM and S-EK wrote the main manuscript text. B-KM, H-SK, and KC performed the experiments. S-EK, H-SK, YK, M-HA, and B-KM analyzed the data and reviewed the manuscript. All authors contributed to the article and approved the submitted version.

## Supplementary material

Supplementary Figure 1 Topographical distribution of EEG theta and alpha activity during the task. Grand-averaged topographies represent the EEG theta (3–8 Hz) and alpha (8–13 Hz) power (normalized *Z*-scores) in both pre- and post-treatment during **(A)** the first half (−2,000 to −1,000 ms before the test-stimulus presentation) and **(B)** the second half (−1,000 to 0 ms before the test-stimulus presentation) of the retention periods in both the tACS-treated and sham groups. The view of the topography is from the vertex, with the nose at the top of the image.
