# Supplementary figures and images for: Neurodynamic correlates for the cross-frequency coupled transcranial alternating current stimulation during working memory performance
Source: Front Neurosci. 2022 Oct 3;16:1013691. doi: 10.3389/fnins.2022.1013691 (PMC9574066; doi:10.3389/fnins.2022.1013691)

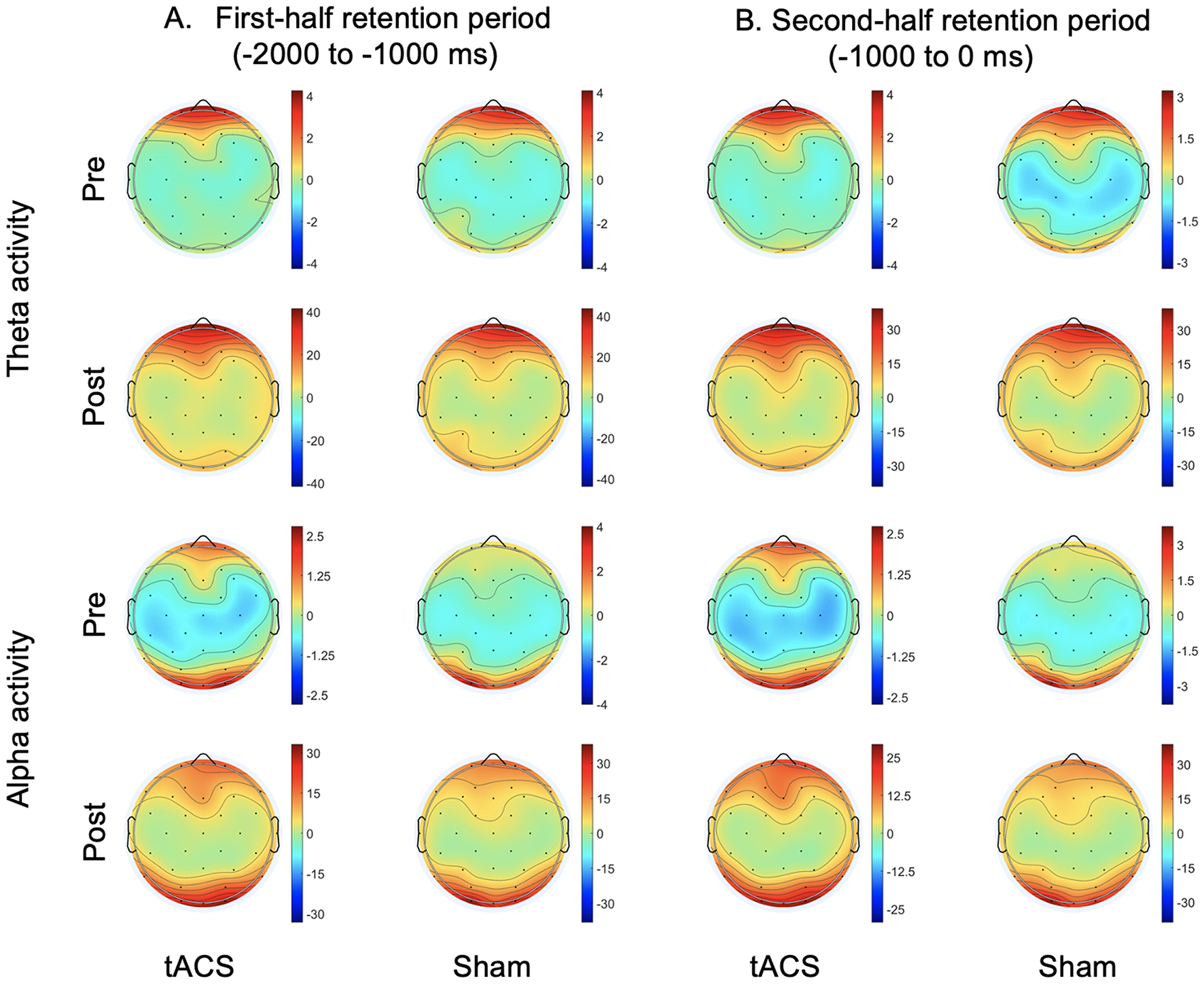

Supplement: Supplementary Figure 1 — Topographical distribution of EEG theta and alpha activity during the task. Grand-averaged topographies represent the EEG theta (3–8 Hz) and alpha (8–13 Hz) power (normalized Z-scores) in both pre- and post-treatment during (A) the first half (−2,000 to −1,000 ms before the test-stimulus presentation) and (B) the second half (−1,000 to 0 ms before the test-stimulus presentation) of the retention periods in both the tACS-treated and sham groups. The view of the topography is from the vertex, with the nose at the top of the image. [file Image_1.TIF]
